# Genome-wide identification and analysis of GRAS transcription factors in the bottle gourd genome

**DOI:** 10.1038/s41598-020-71240-2

**Published:** 2020-08-31

**Authors:** Navjot Singh Sidhu, Gomsie Pruthi, Sahildeep Singh, Ritika Bishnoi, Deepak Singla

**Affiliations:** School of Agricultural Biotechnology, Punjab Agricultural University, Ludhiana, 141004 India

**Keywords:** Data mining, Computational biology and bioinformatics, Plant sciences, Phylogenetics

## Abstract

GRAS genes belong to the plant-specific transcription factors (TF’s) family that are known to be involved in plant growth and development. In this study, we have identified 37 genes from the bottle gourd genome that encodes for GRAS TF’s. Except for the SCLA, we were able to identify at least one gene from each of the 17 subfamilies. Gene structure and chromosomal analysis showed that maximum seven genes are present on Chr7 followed by six genes on Chr1. The subcellular location analysis revealed that most of the genes were localized in the nucleus, except for a few in chloroplast and mitochondria. Additionally, we have identified one tandem gene duplication event on Chr7 and three major motifs that were present in all the GRAS genes. Furthermore, the protein–protein interaction prediction and gene expression analysis showed five candidate hub-genes interact with various other genes and thus probably control the expression of interacting partners in different plant tissues. Overall, this study provides a comprehensive analysis of GRAS transcription factors in bottle gourd genome which could be further extended to other vegetable crops.

## Introduction

Transcription factors (TFs) are the class of proteins that control the functioning of various genes by binding with their promoters and thus involved in the gene regulation process^1^. Previously, more than 320K TFs belonging to 58 transcription factor families have been reported from 165 different plant species. Out of these, GRAS represents one of the major families that are involved in plant growth, development, cell signaling, and stress tolerance^2^. GRAS family was first reported in bacteria and characterized by the three TFs i.e. (i) GAI (gibberellic-acid insensitive); (ii) RGA (repressor of GAI), and (iii) SCR (scarecrow) with the size range from 400 to 770 amino acids^3,4^. It was observed that GAI and RGA are the part of DELLA proteins that take part in gibberellin (GA) and Jasmonate (JA) response as well as light signaling. Likewise, SCR and Short Root (SHR) played a key role in the radial organization of root by forming the SCR/SHR complex^5^.

Previously, it has also been reported that the C-terminal region of GRAS members is highly conserved, while the N-terminal is highly divergent that might provide the specificity to each protein^6^. Furthermore, the evolutionary analysis suggested the phenomenon of horizontal gene transfer (HGT) from bacteria to plants. GRAS gene family is further categorized into different subfamilies such as eight in Arabidopsis and rice^7^, while 8 to 13 in tomato, popular, castor beans, etc^8–10^. Recently, Cenci et al*.* classified the GRAS family into 17 subfamilies such as DELLA, Lateral suppressor (LS), Hairy meristem (HAM), and SCR^11^. Previous studies showed that it is one of the widely explored transcription factor family in the various plant species including tomato, potato, buckwheat, and sweet orange^12–14^.

Bottle gourd (*Lagenaria siceraria*), a member of the Cucurbitaceae family is commonly cultivated in the tropical and subtropical regions and is believed to be originated in southern Africa^15^. It is a diploid species (2n = 2× = 22) having 22 chromosomes that belong to the genus *Lagenaria*. In 2017, the first draft genome of *Lagenaria siceraria* cultivar USVL1VR-Ls was reported with a total of 22,472 genes covering 313.4 Mb region of the genome. In the past, genome-wide analysis studies such as identification of graft responsive mRNA, miRNA has been done in bottle gourd^16,17^. But, in-depth genome-wide analysis of the GRAS transcription factor family is still lacking in case of the bottle gourd. Therefore, considering the use of bottle gourd as an important rootstock material and GRAS transcription factors in plant growth and development, a comprehensive genome-wide search was done in bottle gourd genome to identify the GRAS TFs, their phylogenetic relationship and expression pattern in the different plant tissues.

## Material and methods

The genome and proteome of bottle gourd (*Lagenaria siceraria cultivar USVL1VR-Ls*) was downloaded from the cucurbits genomics database (CuGenDB,  https://cucurbitgenomics.org/)^15,18^. The hidden markov model (HMM) profile of GRAS TF (PF03514.14) was downloaded from the Pfam database using HMMer software^19,20^. We have used PfamScan, InterproScan, and HMMScan to identify the GRAS transcription factors [Suppl. Figure-S1]^20,21^. PfamScan was used to search the complete proteome against the HMM profile at e-value and domE value cut-off 1e^−03^. Similarly, hmmscan was used with incdomE value cut-off 1e^-03^. InterproScan was used against the complete Pfam database and thereafter hits containing GRAS domain (PF03514) were filtered out^22^. SMART tool (https://smart.embl-heidelberg.de/) was used to further confirm the presence of GRAS domain in the respective hits^23^.

### Gene structure, chromosomal location, and gene duplication

To determine the chromosomal location of the GRAS TFs containing genes, nucleotide sequence of respective hits were extracted and subjected against the bottle gourd genome using Blast software^24^. To identify the tandem gene duplication, DupGen_finder software was used which requires the blast results and GFF file of target and out-group species^25^. In this study, BlastP tool was used with the parameters i.e. e-value (1e^−10^), maximum target hits (5) and *A. thaliana* as an outgroup species.

### Sequence analysis and subcellular location prediction

ProtParam and BUSCA webserver was used for computing the protein properties and subcellular location of the GRAS genes^26,27^. To identify the motifs signature present in the GRAS genes, MEME suite (Multiple Em for Motif Elicitation) webserver was used with the maximum number of motifs (10), minimum motif length (6), maximum motif length (50), minimum (2) and maximum (37) sites per motif respectively^28^.

### Phylogenetic tree

We have considered 397 GRAS TF’s from eight different species that were classified into 17 different subfamilies^11^. A local Blast database was constructed and each of the 37 identified GRAS genes were subjected to blast search against this database. Based on the best-hit, each gene was further assigned to the respective group^11^. Subsequently, the final dataset of 434 genes was used to perform multiple sequence alignment (MSA) using Muscle tool^29^ and MEGA7 was used to construct the phylogenetic tree using the JTT model with gamma distribution and complete deletion of removal or gaps^30^. Finally, the tree was visualized using the iTOL (interactive tree of life) software^31^.

### Protein–protein interaction (PPI) prediction and differential gene expression (DGE)

Bottle gourd and *Cucumis melo* (*C. melo*) are phylogenetically closely related species and belong to the same Cucurbitaceae family therefore, we have used *C. melo* as a reference to search GRAS genes and their interacting partners in the String database^32^. All the identified interacting partners were collected and queried against the bottle gourd genome at e-value 1e^−10^ using blast software. The single best-hit for each gene was considered for the construction of a PPI network using Cytoscape^33^. Finally, the top five hub genes from the interaction network were predicted using cytoHubba plugin of Cytoscape^34^. Further, to understand the contribution of these genes, transcriptome of the different tissues from the bottle gourd were searched. We found one dataset in the Cucurbitaceae genome database with the gene expression (FPKM) values for five different tissues. FPKM value of all the 37 GRAS genes were extracted and plotted in the form of a heatmap to understand the relationship.

## Results

### Identification of GRAS transcription factors

**Figure 1:**
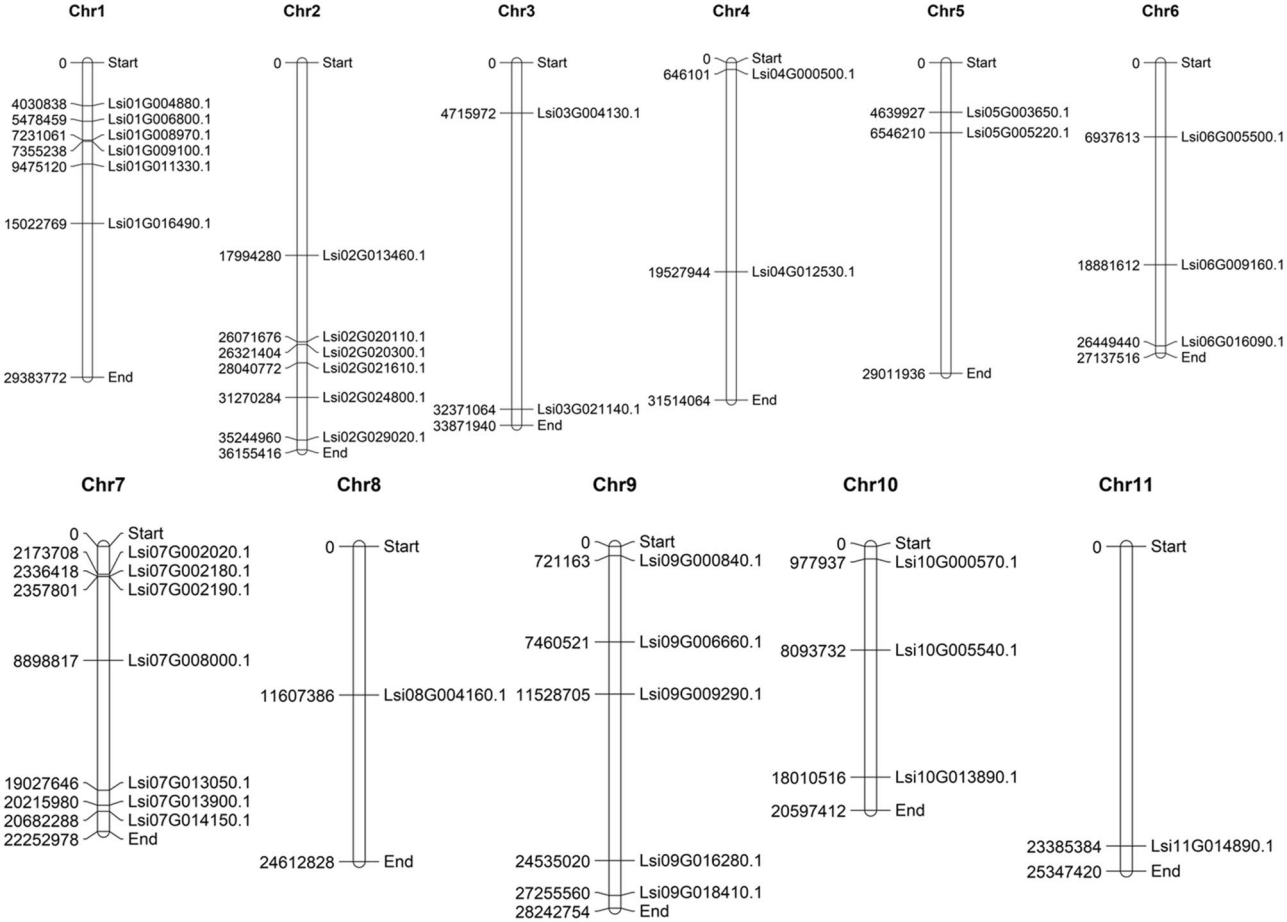
Chromosomal distribution of GRAS genes in bottle gourd using MapChart tool.

We have identified 38, 37 and 37 genes encoding for GRAS TF’s using HMMScan, PfamScan, and InterProScan respectively. Further, domain-based analysis using the SMART tool confirmed the presence of 37 genes from the GRAS family. Therefore, we have finally selected these 37 genes for further in-depth analysis. In addition to the GRAS domain, we identified a DELLA domain in four genes, WD40 in one gene, and a maximum of nine low complexity regions (Table 1). The search for the chromosomal location has identified a minimum of one gene on chromosome-8 and 11, while a maximum of seven genes have been found on chromosome-7 (Figure 1).

**Table 1:** Depict the GRAS genes, their chromosomal location, and additional domains in Bottle Gourd.

S. no	SEQUENCE ID	Chr. No	Start	End	Strand	Domain	LC region	HMM Start	HMM End
1	Lsi08G004160.1	Chr08	11,607,386	11,608,828	( −)	–	1	2	373
2	Lsi03G004130.1	Chr03	4,715,972	4,718,910	( +)	–	2	1	374
3	Lsi02G013460.1	Chr02	17,994,281	17,996,374	( −)	–	2	3	373
4	Lsi01G016490.1	Chr01	15,022,769	15,026,442	( +)	–	9	2	374
5	Lsi06G009160.1	Chr06	18,881,611	18,883,212	( −)	DELLA	1	1	374
6	Lsi09G009290.1	Chr09	11,528,705	11,530,504	( +)	DELLA	1	1	374
7	Lsi09G006660.1	Chr09	7,460,521	7,462,281	( −)	DELLA	3	1	374
8	Lsi09G000840.1	Chr09	721,163	722,962	( −)	–	3	3	374
9	Lsi05G005220.1	Chr05	6,546,210	6,549,403	( +)	–	1	2	373
10	Lsi05G003650.1	Chr05	4,639,927	4,641,627	( +)	DELLA	1	1	374
11	Lsi06G005500.1	Chr06	6,937,613	6,939,013	( −)	–	0	1	374
12	Lsi04G012530.1	Chr04	19,527,943	19,530,358	( +)	–	2	1	373
13	Lsi10G013890.1	Chr10	18,010,515	18,012,671	( +)	–	3	1	373
14	Lsi11G014890.1	Chr11	23,385,384	23,388,109	( +)	–	0	2	373
15	Lsi03G021140.1	Chr03	32,371,065	32,375,712	( +)	–	5	1	374
16	Lsi07G013900.1	Chr07	20,215,979	20,217,644	( −)	–	0	1	373
17	Lsi07G013050.1	Chr07	19,027,646	19,030,122	( −)	–	6	2	374
18	Lsi07G014150.1	Chr07	20,682,288	20,684,252	( +)	–	2	1	373
19	Lsi04G000500.1	Chr04	646,101	648,132	( −)	–	0	1	373
20	Lsi02G024800.1	Chr02	31,270,283	31,271,992	( −)	–	0	2	374
21	Lsi02G021610.1	Chr02	28,040,772	28,042,145	( +)	–	0	1	374
22	Lsi02G020110.1	Chr02	26,071,675	26,073,387	( −)	–	1	2	374
23	Lsi02G029020.1	Chr02	35,244,960	35,246,624	( −)	–	1	3	374
24	Lsi09G016280.1	Chr09	24,535,021	24,537,267	( +)	–	6	1	373
25	Lsi01G009100.1	Chr01	7,355,238	7,356,674	( +)	–	0	4	374
26	Lsi01G008970.1	Chr01	7,231,061	7,232,539	( +)	–	2	2	373
27	Lsi01G011330.1	Chr01	9,475,120	9,476,619	( +)	–	1	2	374
28	Lsi01G006800.1	Chr01	5,478,459	5,480,288	( −)	–	1	2	374
29	Lsi01G004880.1	Chr01	4,030,838	4,032,487	( −)	–	1	3	374
30	Lsi09G018410.1	Chr09	27,255,560	27,257,653	( +)	–	2	3	374
31	Lsi07G002190.1	Chr07	2,357,801	2,359,429	( +)	–	2	3	374
32	Lsi07G002180.1	Chr07	2,336,418	2,338,019	( +)	–	1	3	374
33	Lsi07G008000.1	Chr07	8,898,817	8,900,151	( −)	–	2	2	374
34	Lsi07G002020.1	Chr07	2,173,708	2,175,321	( −)	–	2	1	374
35	Lsi10G000570.1	Chr10	977,937	979,541	( +)	–	0	4	373
36	Lsi10G005540.1	Chr10	8,093,732	8,098,025	( −)	–	3	1	374
37	Lsi06G016090.1	Chr06	26,449,440	26,462,434	( +)	WD40(6), PfamATG16	3	–	–

### Analysis of gene structure, duplicated genes and sequence properties

As observed from the Table 2, the GARS family protein sequence length varies from 378 to 1466 AA’s with a PI range from 4.7 to 8.22. Analysis of the gene structure revealed that out of the 37 genes, 25 genes were encoded by a single exon. However, one gene (Lsi06G016090.1) of length 1466 amino acid was encoded by the 13 exons (Table 2). We have searched for gene duplication events and observed one tandem duplication at Chr7 in the gene Lsi07G002180.1 and Lsi07G002190.1. Further, the prediction of their subcellular location showed that most of the genes were confined into the nucleus, whereas 10 genes in the chloroplast, one each in mitochondria, endomembrane system, and the extracellular space. Further, we have identified 10 most prominent motifs in the GRAS genes with motif length varying from 16 to 41 amino acids [Suppl-1.docx: Table-S1]. Except for the Lsi07G013900 gene, the predicted motifs were localized in the C-terminal region of the genes. We observed that three motifs of length 21, 21 and 25 respectively were highly conserved and present in all the GRAS genes [Suppl.-1.docx].

**Table 2:** Details of GRAS gene properties and their predicted subcellular localization.

S. no	Gene ID	Length	No. of Exon	Mol. Wt	Isoelectric point (PI)	Instability Index	Localization
1	Lsi01G004880.1	549	1	61,188.15	5.99	50.64	Nucleus
2	Lsi01G006800.1	609	1	67,019.36	4.76	52.66	Nucleus
3	Lsi01G008970.1	492	1	55,593.2	5.38	50.84	Chloroplast
4	Lsi01G009100.1	478	1	53,613.45	6.06	42.89	Chloroplast
5	Lsi01G011330.1	499	1	57,309.64	4.7	48.36	Nucleus
6	Lsi01G016490.1	860	2	92,819.33	6.11	55.78	Nucleus
7	Lsi02G013460.1	697	1	79,175.86	6.29	52.51	Nucleus
8	Lsi02G020110.1	570	1	64,176.52	4.83	49.72	Nucleus
9	Lsi02G021610.1	457	1	50,893.89	5.52	43.42	Nucleus
10	Lsi02G024800.1	569	1	65,098.16	5	50.28	Nucleus
11	Lsi02G029020.1	554	1	62,181.42	7.3	47.3	Nucleus
12	Lsi03G004130.1	678	2	74,745.31	5.22	60.93	Nucleus
13	Lsi03G021140.1	921	5	103,250.5	6.04	56.83	Chloroplast
14	Lsi04G000500.1	491	3	55,291.75	5.46	55.85	Endomembrane system
15	Lsi04G012530.1	650	4	72,522.1	5.48	51.25	Chloroplast
16	Lsi05G003650.1	566	1	61,666.02	5.45	48.29	Nucleus
17	Lsi05G005220.1	562	3	62,672.06	5.74	49.13	Chloroplast
18	Lsi06G005500.1	466	1	52,413.36	5.99	42.97	Nucleus
19	Lsi06G009160.1	533	1	58,170.78	5.4	49.47	Nucleus
20	Lsi06G016090.1	1,466	13	164,426.8	8.22	51.62	Nucleus
21	Lsi07G002020.1	537	1	58,978.35	4.92	55.18	Nucleus
22	Lsi07G002180.1	533	1	59,559.44	5.91	49.61	Chloroplast
23	Lsi07G002190.1	542	1	60,457.47	6.04	49.28	Chloroplast
24	Lsi07G008000.1	444	1	48,576	5.75	53.95	Chloroplast
25	Lsi07G013050.1	793	3	87,345.74	5.32	59.39	Extracellular space
26	Lsi07G013900.1	378	2	43,730.93	6.71	48.29	Chloroplast
27	Lsi07G014150.1	654	1	71,927.6	5.56	62.52	Mitochondrion
28	Lsi08G004160.1	480	1	53,818.47	5.82	47.97	Nucleus
29	Lsi09G000840.1	599	1	65,260.66	5.97	53.5	Nucleus
30	Lsi09G006660.1	586	1	65,000.5	5.26	44.37	Nucleus
31	Lsi09G009290.1	599	1	65,805.21	5.1	55.37	Nucleus
32	Lsi09G016280.1	748	1	81,440.6	5.91	58.32	Nucleus
33	Lsi09G018410.1	457	2	51,735.59	5.42	47.37	Nucleus
34	Lsi10G000570.1	534	1	59,572.37	5.64	46.02	Nucleus
35	Lsi10G005540.1	517	4	58,862.45	6.2	53.75	Nucleus
36	Lsi10G013890.1	718	1	81,592.12	5.41	48.86	Nucleus
37	Lsi11G014890.1	437	2	49,173.92	6.02	38.31	Chloroplast

### Phylogenetic analysis

**Figure 2:**
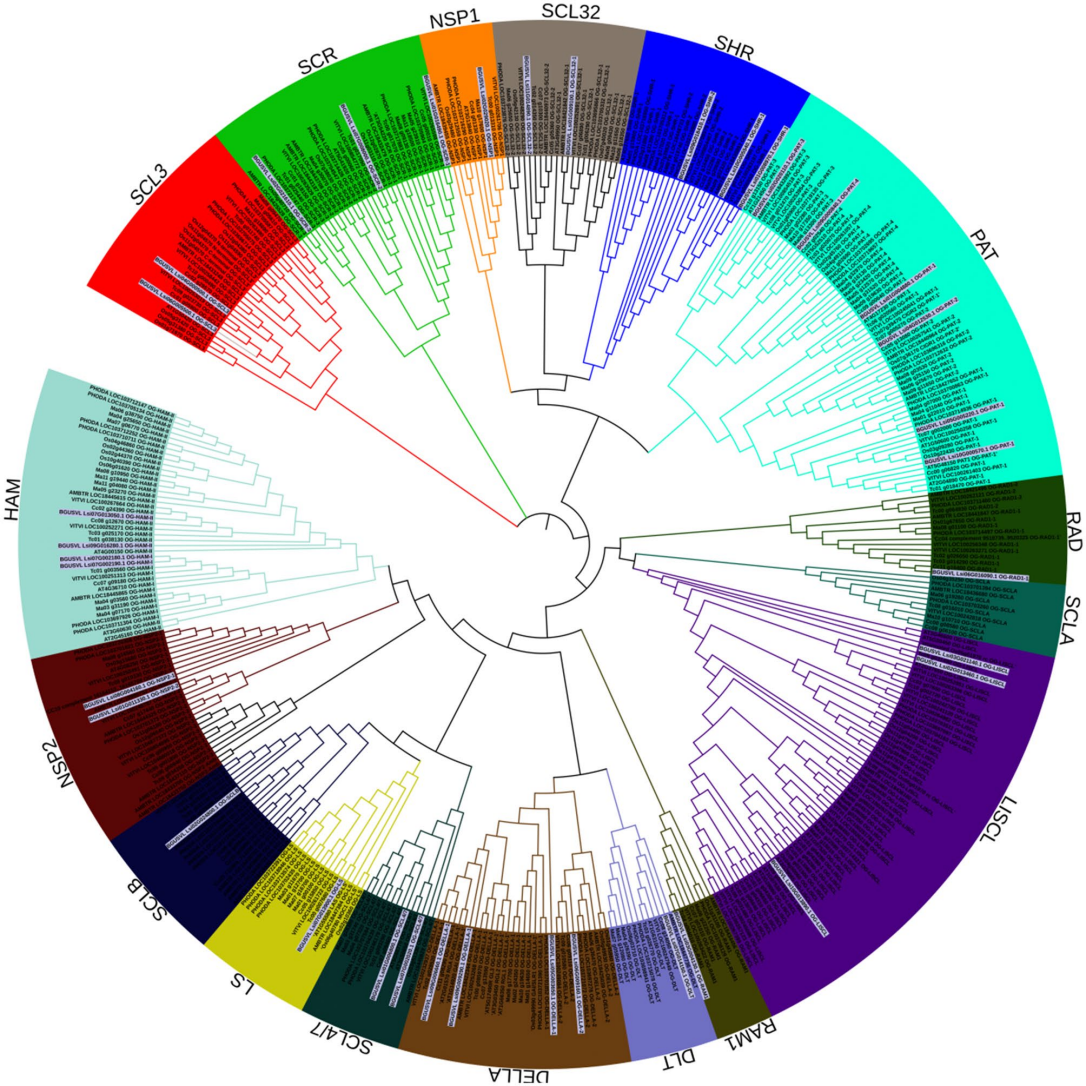
Phylogenetic tree of GRAS sequences visualized using iTOL software with background colors differentiates subfamilies. Bottle gourd genes in the subfamily are highlighted in grey color.

A phylogenetic tree was constructed from the 434 sequences including 397 from previously published work and 37 from the bottle gourd genome. As observed from the Figure-2, all the 37 identified GRAS genes could be divided into 16 different subfamilies. Further analysis revealed that except for the SCLA, at least one gene from each subfamily was present in the genome (Fig. 2). A maximum of six genes were observed from the Phytochrome A signal transduction (PAT) subfamily followed by four genes from the HAM and DELLA subfamilies. In contrast to that, LS and Required for arbuscule development (RAD) belong to the smallest subfamily with only one gene.

### Protein–protein interaction (PPI) prediction and DGE analysis

**Figure 3:**
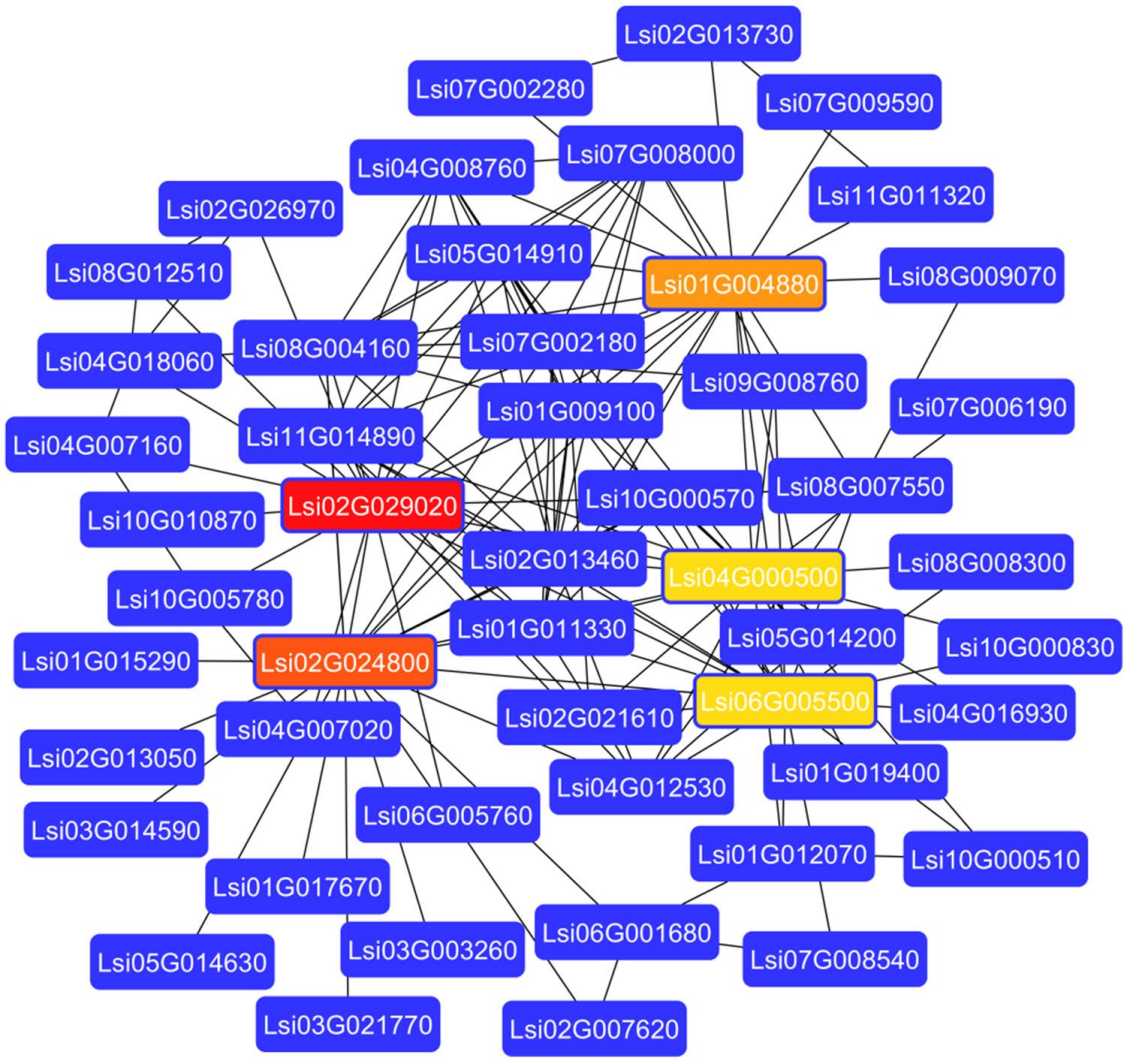
Depicts the predicted protein–protein interactions between GRAS Transcription Factors using Cytoscape.

**Figure 4:**
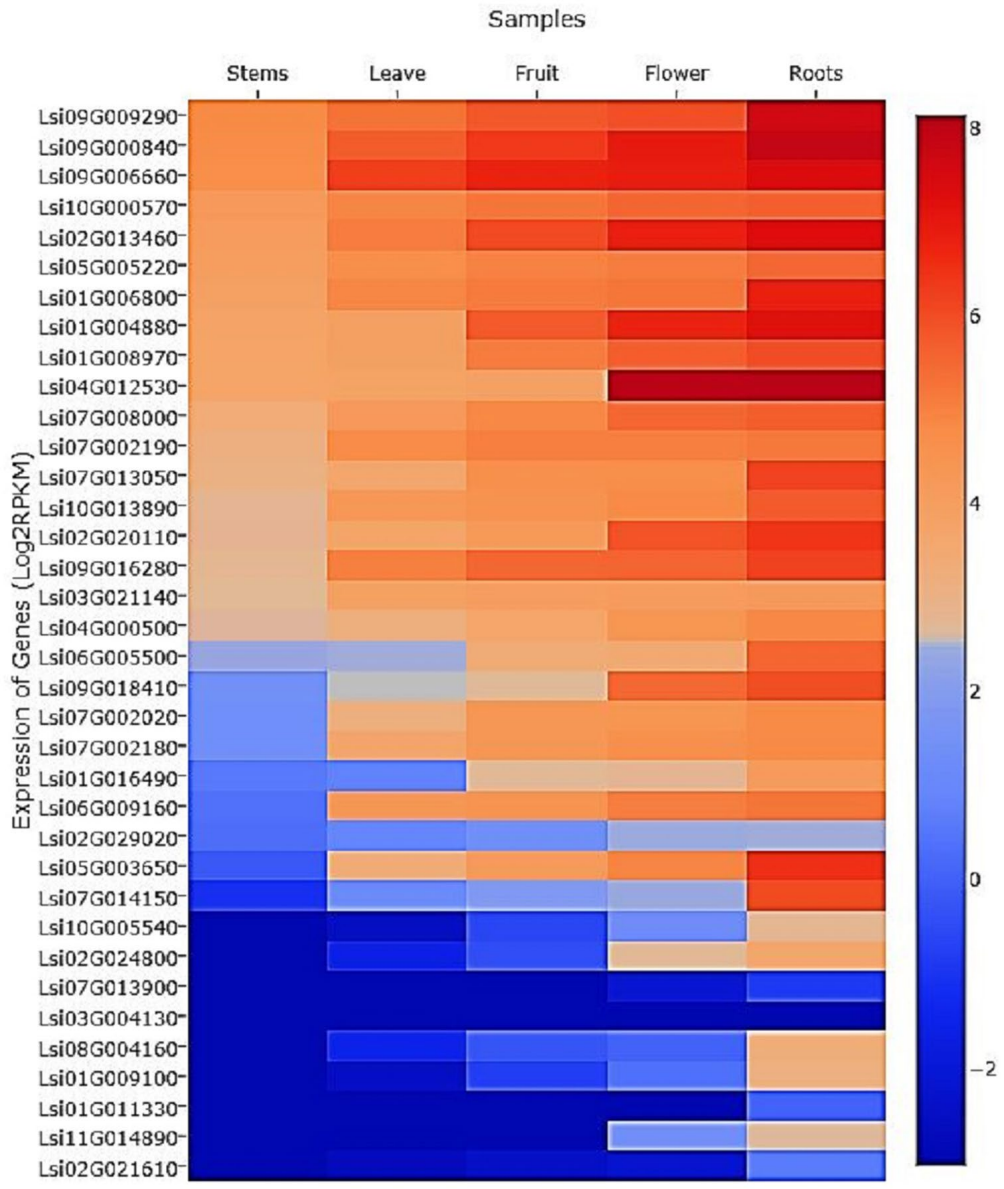
Differential Gene Expression of GRAS transcription factors.

PPI prediction analysis revealed that a total of 178 unique genes from *C. melo* were involved in 467 possible interactions. Next, we searched their homologs in the bottle gourd genome. We observed that two genes (XP_008452266.1 and XP_008459103.1) do not have any homologs, and therefore we excluded them further in our study. Finally, we had a set of 169 exclusive genes from the bottle gourd genome that possibly interacts with each other to control various biological functions. Further, network analysis revealed the presence of 169 nodes and 467 edges with clustering coefficient value 0.452 and 5.4 average no. of neighbour’s, respectively. Based on the maximum degrees, we have identified Lsi02G029020, Lsi02G024800, Lsi01G004880, Lsi04G000500, and Lsi06G005500 as the candidate hub genes with maximum interactions 22, 21, 20, 19, and 19 respectively (Fig. 3). We have also studied expression level of the GRAS transcription factors in different plant tissues and observed that Lsi01G004880 gene was up-regulated in all five tissues with higher expression (> 6 folds) in the root tissue (Fig. 4). Similarly, Lsi04G000500 and Lsi04G005500 expressed in the root tissue, whereas with no or poor expression in stem and leaves. On the other hand, the Lsi02G024800 gene showed down-regulation in the stem and leaves while no significant change in the expression pattern of the Lsi02G029020 gene was observed (Fig. 4). The GRAS genes Lsi05G003650, and Lsi07G014150 expressed in roots but with no significant expression in the stem tissues.

### Functional annotation of Hub and interacting genes

We investigated the function of five candidate hub genes based on their involvement in a biological and cellular process. Lsi02G024800 gene is a DELLA protein that acts as a repressor of GA induced growth and interacts with gene encoding for auxin efflux carrier, gibberellin receptor, and lignin degradation and detoxification. Thus, it eventually controls the root growth, seed germination and elongation of the stem. Similarly, Go-term analysis showed that Lsi02G029020 gene has DNA binding transcription factor activity involved in the regulation of transcription, gene expression and is localized in the nucleus. This gene interacts with transcriptional activator genes that control the genes encoding for stamen development, cell expansion, and flowering time and also modulate the growth of roots. Next, hub-gene Lsi01G004880 is a scarecrow-like protein that interacts with 10 other proteins including a Zn-finger domain and phytohormone protein. The phytohormone gene controls the phototropic response by modulating the light signal. Similarly, the Lsi04G000500 gene also belongs to the scarecrow-like class and interacts with genes like serine/arginine-rich SC35-like splicing factor SCL28, jasmonate O-methyltransferase (JMT), and Nutcracker (NUC). JMC gene converts the jasmonate into methyl-jasmonate and plays an important role in plant defense. NUC gene acts as a transcriptional activator and is involved in the regulation of flowering, and asymmetric cell division. Also, Lsi06G005500 gene belongs to the scarecrow-like class and acts on auxin response factor (ARF), gibberellin 2-beta-dioxygenase-1 (GA2OX1), and phytochrome that is involved in plant growth and development.

## Discussion

GRAS proteins have been recognized as an important plant cellular component that play role in signal transduction process, root, and shoot development^35,36^ as well as in managing the various kind of biotic and abiotic stress^37^. In the present study, we explored the GRAS family in the bottle gourd genome including their gene structure, chromosomal distribution, phylogenetic analysis, and gene expression in different tissues.

In this study, a total of 37 GRAS genes were computationally identified in the bottle gourd genome, which is lower than tomato, Cucurbita but higher than Arabidopsis. Previous studies reported that GRAS genes are mostly encoded by the single exon with a length of around 400–770 amino acids^35^. We have also observed a similar pattern in case of bottle gourd with a small variation in the gene length. Intronless gene is the important feature of prokaryotic genome thus, it suggested the phenomenon of horizontal gene transfer from the prokaryotes as well as the close evolutionary relationship among different members^4^. Further, our subcellular localization analysis is also in agreement with previous studies that most of these genes are localized in the nucleus. We also found one tandem gene duplication event that could play an important role in the expansion of GRAS gene family.

It is well-known that some genes of the DELLA subfamily such as GAI, RGA, and RGL act as repressor of gibberellin signaling, while SCR and SHR are involved in radial root development^5,38,39^. Expression of the DELLA gene (a negative regulator of GA signaling) in multiple tissues highlighted their role in plant growth and developments^40,41^. Similarly, SCL3 is involved in the root elongation, and SCR interacts with the RGA gene that ultimately controls the root meristem size in Arabidopsis^42^. Previous studies reported that mutation in the SCR gene resulted in the disruption of the asymmetrical cell division and thus affect the root growth and development. Thus, a higher expression of the SCR gene in the plant roots is helpful for the radial organization of roots^43^.

Bottle gourd has been widely used as a rootstock in controlling different types of biotic and abiotic plant stress^44–47^. Previous studies suggested that the SCL14 gene in Arabidopsis is essential for the activation of stress-inducible promoters^48^. Likewise, overexpression of VaPAT1 and OsGRAS23 confers the abiotic stress tolerance in Arabidopsis and rice^37,49^. In 2019, Garcia-Lozano et al*.* compared the transcriptome of the bottle gourd grafted on the watermelon and vice-versa^44^. They reported more than 400 mobile RNA between the different hetero-grafts and observed that the use of bottle gourd as rootstock increased the size and rind thickness of the watermelon fruits. Similarly, Liu et al*.* reported the differential expression of 787 genes between watermelon homo and heterograft (bottle gourd rootstock)^16^. To highlight the importance of bottle gourd as rootstock, Wang et al*.* (2020) analyzed the transcriptome of bottle gourd (rootstock) and watermelon (scion) under chilling reatment. They reported that bottle gourd homograft, as well as hetero-graft, are tolerant to chilling stress compared to the water-melon homograft^50^. Thus, in-depth analysis of GRAS transcription factors in the bottle gourd genome will be helpful for enhancing the use of bottle gourd as valuable rootstock material and could also be extended in other vegetable crops.

## Supplementary information

Supplementary Information.
