# Supplementary material for: Genome-wide identification and analysis of GRAS transcription factors in the bottle gourd genome
Source: Sci Rep. 2020 Aug 31;10:14338. doi: 10.1038/s41598-020-71240-2 (PMC7459283; doi:10.1038/s41598-020-71240-2)
Supplement: Supplementary file 1 — Supplementary Information. [file 41598_2020_71240_MOESM1_ESM.docx]

Genome-wide identification and analysis of GRAS transcription factors in the Bottle Gourd genome

Navjot Singh Sidhu^1^, Gomsie Pruthi^1^, Sahildeep Singh^1^, Ritika Bishnoi^1^, Deepak Singla^1^*

^1^School of Agricultural Biotechnology, Punjab Agricultural University, Ludhiana

**
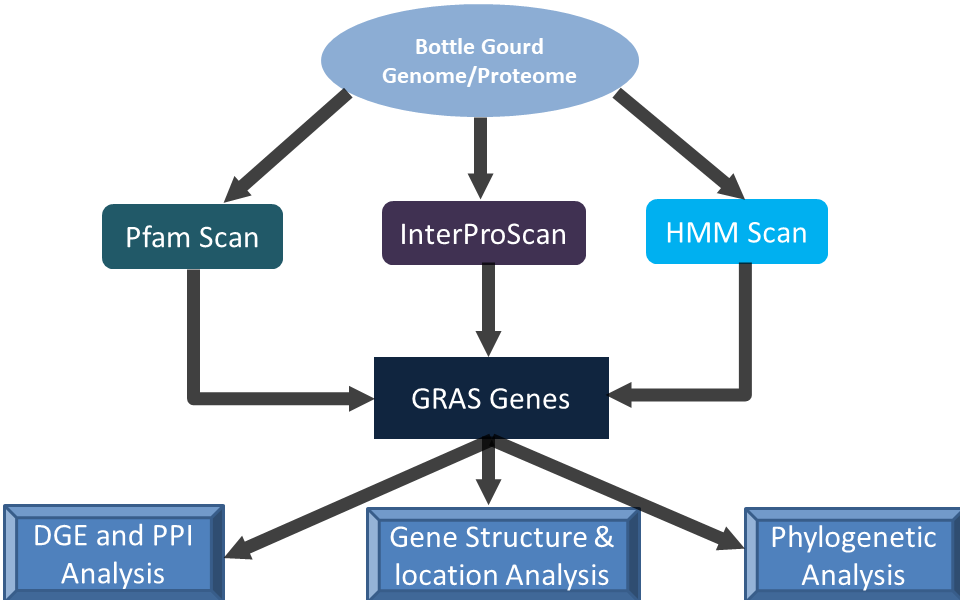
**

**Figure-S1: Depicted the flow diagram of Identification and analysis of GRAS Gene family.**

**Table-S1: Details of the identified Motifs in GRAS genes**

| **Motif** | **Motif Consensus** | **Motif Width** | **No. of Sites** |
| --- | --- | --- | --- |
| Motif-1 | CPYJKFAHFTANQAILEALEGEDRVHIIDFDIMQGL | 36 | 35 |
| Motif-2 | EZEYLGREIVNIVACEGAERVERHETLGKWRSRMEMAGFKP | 41 | 23 |
| Motif-3 | EENGCLVLGWKDRPLVAASAW | 21 | 37 |
| Motif-4 | QEABHNGPSFLTRFVEALHYYSAJFDSLD | 29 | 36 |
| Motif-5 | QWPTLIQALATRPGGP | 16 | 32 |
| Motif-6 | PYGDPMQRLAAYFAEALAARL | 21 | 36 |
| Motif-7 | EGLEZTGRRLAEFAESLGVPFEFNP | 25 | 37 |
| Motif-8 | ARDSLLRLIKSLNPKIVTLVE | 21 | 35 |
| Motif-9 | LLLACAEAVSSNBLELAQELL | 21 | 37 |
| Motif-10 | LRVRPGEALAVNCVLQLHRLL | 21 | 35 |


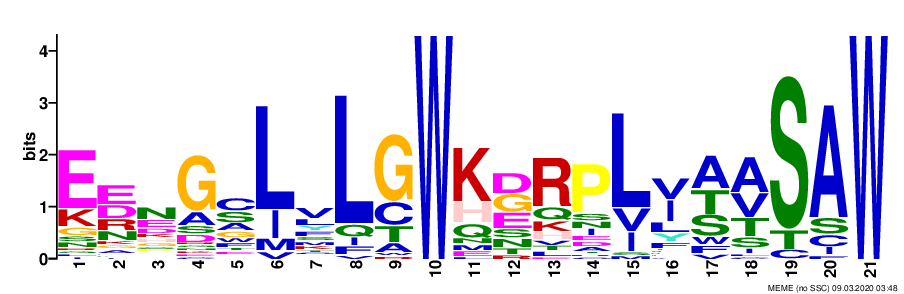


**Figure-S2: Weblogo of the Motif-3 representing the conservation pattern**


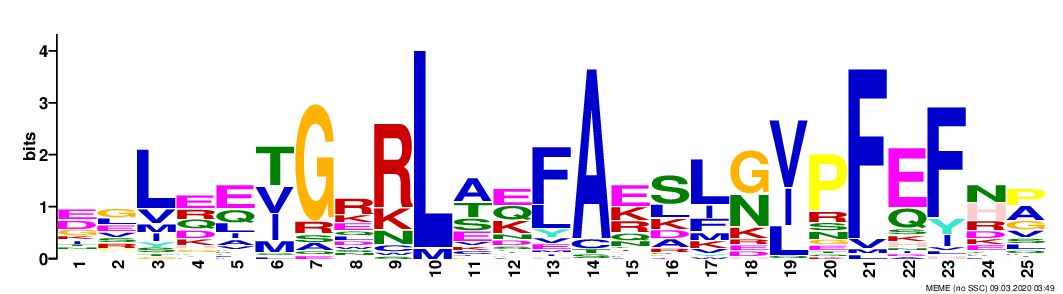


**Figure-S3: Weblogo of the Motif-7 representing the conservation pattern**


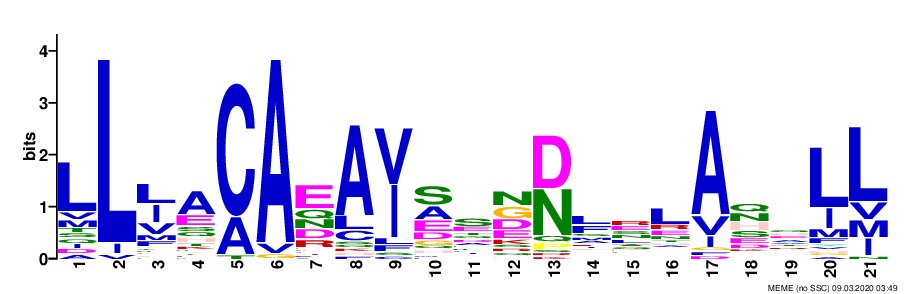


**Figure-S4: Weblogo of the Motif-9 representing the conservation pattern**
